# VACS: VAccination disComfort Scale

**DOI:** 10.3390/clinpract12060110

**Published:** 2022-12-15

**Authors:** Manolis Wallace, Stavros Antonopoulos, Vassilis Poulopoulos

**Affiliations:** 1 ΓAΒ LAB—Knowledge and Uncertainty Research Laboratory, University of Peloponnese, 221 31 Tripoli, Greece; 2 “Agia Sofia” Children’s Hospital, 115 27 Athens, Greece

**Keywords:** scale, vaccination, discomfort, crying

## Abstract

The vaccination of children is a crucial tool to protect both individuals and the world in general from various diseases and pathogens. Unfortunately, the vaccination procedure is not a pleasant one for all children, with many experiencing various levels of discomfort, sometimes reaching intolerable levels. In the first part of this work, we develop VACS, a tool that measures the discomfort children experience during vaccination. VACS takes into consideration the complete timeline of the vaccination experience from the perspective of the child, starting from the moment the child enters the doctor’s office through to their departure, and also the complete range of manifestations of discomfort, ranging from moaning and crying to facial expressions and posture. Their discomfort is quantified as a number from 0 to 25, with zero corresponding to a smooth vaccination and 25 to maximal/unbearable discomfort. In the second part of the work, we apply VACS to 40 vaccinations of children aged 2 to 12. Our findings show that approximately 40% of the children do not face discomfort during vaccination, but for the rest discomfort of varying degrees is observed. We also find that doctors are content with their patients facing considerably higher discomfort levels than what the children themselves are willing to withstand: doctors are content with VACS values up to 19 whilst children start to suffer when the VACS value exceeds 11. Surprisingly, characteristics such as (a) gender, (b) whether the state’s recommended vaccination program has been implemented in full, and even (c) prior negative vaccination experiences are found to be poor predictors of vaccination discomfort. Age on the other hand may be a factor, with younger children experiencing discomfort more often and more intensely; more research is required in order to validate this with higher confidence. The formulation of VACS opens the door for more systematic work towards the mitigation of vaccination discomfort for children.

## 1. Introduction

It is well established that increased vaccine coverage has led to a decline in diseases [1]. Vaccination is a great tool for the protection of society from many viruses that would otherwise continue to have a greater impact. However, more recently vaccination coverage has begun to drop. For example, the global vaccination coverage dropped from 86% in 2019 to 81% in 2021 [2]. For specific diseases and pathogens, the coverage can be significantly lower than that [1], putting individuals and also communities more at danger. Even in privileged “first world” countries that do not face barriers in accessing vaccines, there are cases in which vaccination coverage is less than ideal; indicatively, in at least six states in the U.S.A., vaccination coverage is lower than the threshold that the CDC has set for the prevention of outbreaks [1]. Multiple reasons can lead to a reduced uptake of vaccinations, ranging from the inaccessibility of proper medical care to misinformation and even from concerns about potential side-effects [3] to the peculiarities of the applicable legal framework [4]. Regardless of the reasons, the lowered uptake of vaccines is a very negative development, in view of which every tool that has the potential to enhance vaccination uptake needs to be explored. The assessment of the practice of health care providers during vaccination and the provision of corresponding feedback have been identified as tools that contribute to improved vaccination uptake [5]. Further strengthening this view, in [6] it was observed that influenza vaccine coverage was significantly improved via vaccination-related clinic process changes. Therefore, assessing how vaccines are delivered and making appropriate changes have the potential to affect the long-term uptake of vaccinations, greatly impacting the health both of individuals and of the broader community, now and in the future.

But with needles being a source of discomfort or even fear for so many, the vaccination procedure is not pleasant for all. As immunization schedules around the world typically cover ages from birth to 18 years of age and most vaccinations take place during the first years of childhood, it is particularly young children that suffer through the vaccination procedure. The study in [7] established that needles and blood draws are the primary source of fear when it comes to children and medical procedures, underlining the need for research towards smoother medical operations that involve needles and are provided to children. Vaccination is perhaps the most prominent medical operation in the list, due to its application to all children (not only the ones with some specific condition) and to the fact that it occurs many times throughout childhood.

As an example, the standard immunization schedule in the UK includes 27 vaccinations to be carried out from birth until the age of 15 [8], mostly delivered via needles with the rotavirus vaccine being the exception as it is delivered through the mouth. That is a very large number of vaccination shots to go through for those children for whom the vaccination experience is a particularly negative one. Whilst there are small differences in the immunization schedules between different countries, the large number of vaccination shots from infancy until puberty is a common characteristic.

There is growing evidence that the discomfort children experience during vaccination and other procedures involving needles is not a given but can be mitigated via adaptations of the procedure. In [9] for example, we see that even without any change in the medical procedure, just by changes in the parents’ behavior, the children’s needle-related distress is affected. Similar findings are reported in [10] for babies, with the caveat that the study only considers pain and ignores other forms of distress. Moving in the same direction, in [11] we see that trust between the health care provider and the child can reduce needle phobia. Thus, taking steps to establish or enhance this trust can also mitigate the phobia. We should note, though, that the work in that article was based on interviews of the clinicians regarding their overall opinions, without examining specific children/cases and quantifying their observations. Thus, the influence of perception bias on the drawn conclusions cannot be excluded. Moreover, the conclusions are only qualitative, with no quantifiable assessment of the extent of influence that trust can have on the mitigation of needle phobia. Still, to this day we have mainly taken this discomfort for granted and forced our children to push through it, without giving any more consideration to it.

Of course, it is not fair to say that the issue of reducing children’s distress towards needles has not been examined at all. However, more can be done. In [12], for example, we have a recent survey on doctors’ preferred approaches to reducing children’s distress towards needles. What is unfortunately missing is the consideration of the children’s views. Thus, there is an inherent bias in the approach, as it looks at what the doctors assume reduces children’s distress without also taking the steps required to assess whether the doctors’ preferred approaches have indeed the desired outcome.

The study in [13] makes an attempt to consider the vaccination distress experienced by infants and preschoolers. The weakness in that study is that the nature and different manifestations of distress were not examined. Instead the MBPS [14] scale was used to assess distress for infants and the FLACC [15] scale was to assess distress for preschoolers. Both scales focus strictly on pain during and post-operations, ignoring other manifestations and elements of discomfort, such as fear leading up to a medical operation.

In this work we aim to address this gap in theory and practice, by seeing the vaccination procedure from the perspective of the children and providing a tool to not only assess by also quantify children’s discomfort during vaccination. We develop a scale that takes into consideration the complete timeline of the vaccination experience from the perspective of the child, starting from the moment the child enters the doctor’s office up until their departure, and also the complete range of manifestations of discomfort, ranging from moaning and crying to facial expressions and posture.

More specifically, we develop a new scale, VAccination disComfort Scale (VACS), that is intended to measure children’s discomfort during vaccination. VACS is based on doctor’s observations before, during and after vaccination. It adds less than one minute to the overall duration of the vaccination procedure and produces a number in the range of 0–25, with 0 indicating total comfort and 25 indicating maximal discomfort. Measuring discomfort is (1) a first step towards gaining a deeper understanding of who experiences discomfort and to what extent, but also (2) a tool for assessing which vaccination procedures are the most pleasant for children. With young children making up a quarter of the world’s population [16], any intervention that affects the quality of their experience in a procedure as frequent as children’s vaccination has the potential to make a huge difference in their quality of life. At least this is our hope.

The remaining of this article is organized as follows: In Section 2 we develop VACS and present the methodology we have followed in order to validate it. In Section 3 we present the findings of our clinical trial, while in Section 4 we elaborate further on our findings, discuss the implications of our work and identify weakness and potential future directions. Finally, Section 5 lists our concluding remarks.

## 2. Materials and Methods

### 2.1. VACS Structure

In order to measure children’s discomfort during vaccination in the most objective way possible, we base our calculations on the doctor’s observations. The patient is observed from the moment they enter the room or area where the vaccination takes place until they leave and a number of parameters indicating distress and discomfort are noted.

Recorded observations come from four distinct stages:

1. Stage I—Entrance. How the child is/behaves when entering the examination area;
2. Stage II—Examination. How the child is/behaves while being examined by the doctor and building up to the actual examination;
3. Stage III—Procedure. How the child is/behaves during the actual medical procedure of vaccination;
4. Stage IV—Completion. How the child is/behaves when the actual vaccination has been completed.

Weights are assigned to each parameter, so that a single overall value of discomfort can be computed.

### 2.2. VACS Parameters

#### 2.2.1. Crying

Crying is of course a major indication of discomfort during vaccination, especially for younger children. It can be an indicator not only of pain but also of fear or other types of distress. That is why it is not only observed during/after the vaccination shot but very often even before that. The extent of crying can vary and this can be indicative of the level of discomfort. In order to take the extent of crying into account, we use the following levels:

1. No crying;
2. Light moaning, or intermitted crying;
3. Loud crying, constant howling or sobbing.

#### 2.2.2. Hesitation

Hesitation (or the lack of it) is observed as the child enters the vaccination room/area. Hesitation is a key element that can at times even escalate to a total refusal to enter the doctor’s office. The obvious cases are:

1. No hesitation;
2. Hesitation.

#### 2.2.3. Activity

The child’s activity at various stages of the procedure is a key element that may or may not coexist with other observed parameters. Three distinct levels are considered:

1. Relaxed posture, in which the child is relaxed, lying down or in another position that is normal for the child’s age and moves easily;
2. Twisting, moving back and forth, generally being in tension;
3. Assuming a defensive of fetal position, or being rigidly fully stretched.

#### 2.2.4. Facial Expressions

Facial expressions are great indicators of how one feels and could not possibly be left out in this work. Three categories are identified as follows:

1. Relaxed muscles, smiling, showing comfort;
2. Occasional grimaces with tight facial muscles, furrowed brow, chin or jaw;
3. A continuous grimace, frequent or constant chin shaking, clenched jaw.

#### 2.2.5. Support

We observe the support that the child may need in order to calm down, as follows:

1. Child is asleep or child is awake but content and relaxed;
2. The child whines but can be calmed by touching, hugging or talking;
3. The child remains inconsolable no matter what.

#### 2.2.6. Cooperation

The way the child (eventually) cooperated for the vaccination to take place is a great indicator of the level of discomfort. We classify cooperation based on who was required to be physically involved to help (or potentially restrain) the child for the vaccination to take place, as follows:

1. The child makes it on their own;
2. The parent/guardian needs to be involved;
3. The parent/guardian and also the clinic’s staff need to be involved;
4. The parent/guardian is asked to step back, the clinic’s staff take over on their own and restrain the child as needed.

### 2.3. VACS Calculation

The four stages that we are considering (entry, examination, procedure and completion) do not all have the same importance when discussing vaccination discomfort. It is the time leading up to vaccination and the actual act of vaccination that cause the most discomfort to children. This is reflected in the distribution of the scores for the different stages of VACS; as can be seen in Table 1, the most points are awarded for observations during the examination and procedure stages, whilst the stage when the vaccination has been completed carries the least weight.

For all of the examined parameters, the first category listed in the previous subsection is the one indicating no discomfort and therefore does not contribute any score to the VACS measurement. The other categories are scored as follows:

#### 2.3.1. Stage I—Entrance

Hesitation to enter the vaccination area is scored with 3 points. Light intermitted crying is scored with 1 point whilst loud constant crying contributes 2 points, reaching a maximum of 5 points for the entrance stage.

#### 2.3.2. Stage II—Examination

Occasional grimaces are assigned 1 point and a constant grimace is scored with 2 points. Light intermitted crying is scored with 1 point whilst loud constant crying is scored with 2 points. Extensive activity is assigned 2 points and defensive/fetal position is assigned 4 points, reaching a maximum of 8 points for the examination stage.

#### 2.3.3. Stage III—Procedure

Being agitated but able to calm down scores 1 point while being inconsolable scores 2 points. Requiring the assistance of a parent or guardian to complete the vaccination scores 1 point. 3 points are given if the staff need to be involved and 4 points if the parent/guardian needs to step away for the staff to take over alone. Light intermitted crying is scored with 1 point whilst loud constant crying is scored with 2 points, reaching a maximum of 8 points for the procedure stage.

#### 2.3.4. Stage IV—Completion

Light intermitted crying is scored with 1 point whilst loud constant crying is scored with 2 points. Extensive activity is assigned 1 point and the defensive/fetal position is assigned 2 points, reaching a maximum of 4 points for the examination stage.

**Figure 1:**
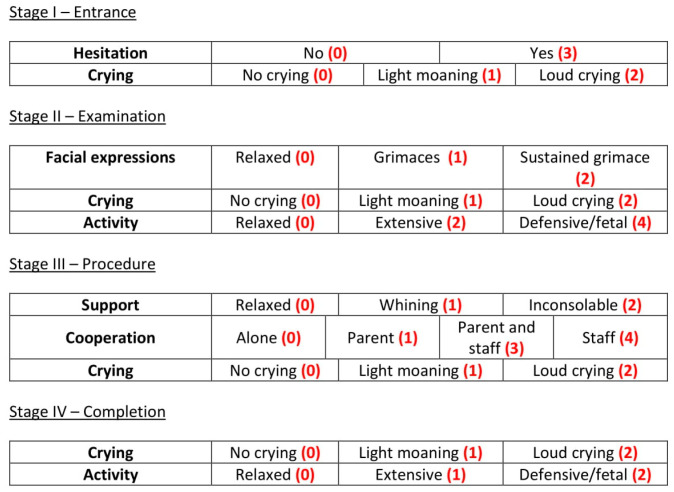
The weights of parameters considered in VACS.

The weights are summarized in Figure 1, for easy reference.

### 2.4. Clinical Settings

Having described VACS, one question is that of validation, i.e., the examination of whether larger VACS values truly correspond to greater discomfort. Another question is that of the establishment of the most suitable thresholds in order to interpret VACS values. In other words, establishing what is a typical VACS measurement and which measurement should be considered as excessive. To answer these questions, we have applied VACS in a clinical setting. Participants were recruited using convenience sampling. Specifically, all children visiting one of the pediatric clinics participating in this research were considered. The participating clinics were a public one and two private ones, each with its own pediatrician in charge. The two inclusion criteria were:

1. The child being between 2 and 12 years;
2. The accompanying parent/guardian being sufficiently fluent in Greek in order to provide written informed consent.

Once consent was acquired, the doctor used the form presented in Appendix A in order to log the observations needed to calculate VACS. Making the observations required to fill in the form did not interfere with the doctor’s work or alter the vaccination procedure in any way. In fact, the forms were actually filled in by the doctor after the patient’s visit was over, therefore the study did not affect the observed vaccination procedure in any way.

In addition to the VACS parameters, the form also records the doctor’s, parent/guardian’s and child’s own assessment of the vaccination experience. Doctors and parents/guardians are directly asked to distinguish between smooth, acceptable and bad procedures. Children’s feelings on the other hand are probed indirectly by asking whether they are willing to return to the doctor’s office for a future vaccination. Parents/guardians are also asked to report whether the child has had a prior bad vaccination experience or vaccination side effect.

The study’s protocol has been assessed and approved by the Research Ethics Committee of the University of Peloponnese (protocol code 20836/14 September 2022)

## 3. Results

### 3.1. Clinical Data

Following the procedure described in Section 2.4, the vaccinations were carried out by three pediatricians in one public and two private pediatric clinics and their VACS parameters were recorded. The pediatricians submitted forty-five records of vaccinations, of which five were discarded after a second check as the children involved were found to be outside the 2–12 year age range for the inclusion criterion; one was a newborn and four were 13 years old or older. Thus 40 vaccination records were ultimately considered in this study.

The mean age of the participants was 8.79 years old, ranging from 2 years 0 months 4 days old to 12 years 6 months 25 days old. Twenty-five of the participants were boys (62.5%) and fifteen were girls (37.5%). Thirty-one (77.5%) of the children had completed their recommended vaccination schedule while nine (22.5%) of the children had skipped one or more of the vaccines recommended for their age group.

**Figure 2:**
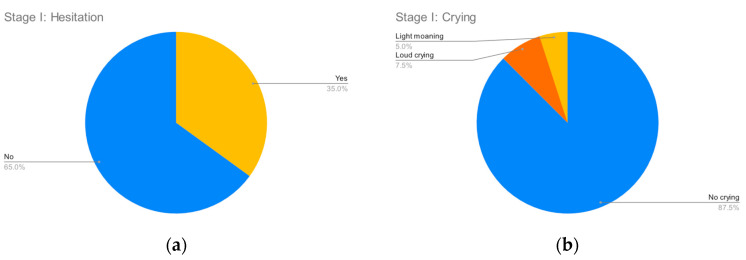
Stage I parameters. (**a**) Hesitation; (**b**) Crying.

**Figure 3:**
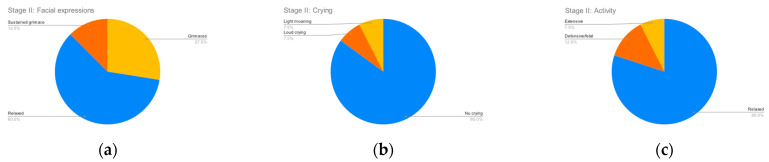
Stage II parameters. (**a**) Facial expressions; (**b**) Crying; (**c**) Activity.

**Figure 4:**
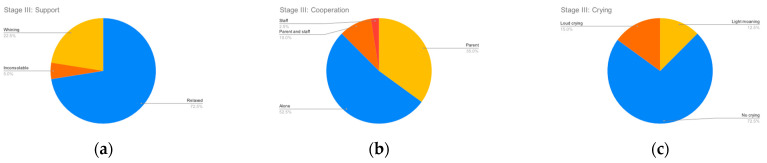
Stage III parameters. (**a**) Support; (**b**) Cooperation; (**c**) Crying.

**Figure 5:**
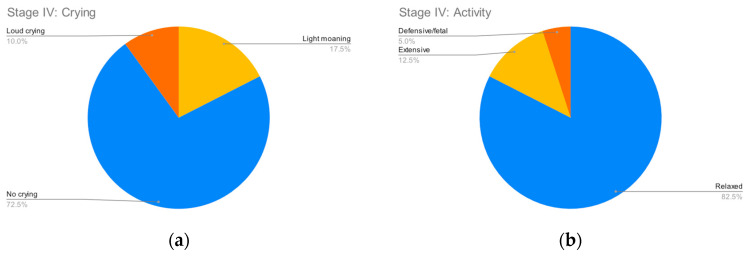
Stage IV parameters. (**a**) Crying; (**b**) Activity.

Figure 2, Figure 3, Figure 4 and Figure 5 summarize the records for each of the observed parameters. To facilitate reading in all graphs, a blue color has been assigned to observations that do not indicate any discomfort, followed by yellow, orange and red in order of the severity of the observed behavior. The most common discomfort indicator, observed in nineteen (47.5%) of the cases, is a lack of cooperation in stage III, whilst the least common one is crying in stage I, observed in five (12.5%) of the cases.

Doctors found the vaccination procedures ran less than smoothly in three (7.5%) of the cases, while on the other hand children in six (15%) of the cases found the procedure so discomforting that they are reluctant to return for a future vaccination.

For seven (17.5%) of the children, their parents/guardians reported a prior bad vaccination experience. No prior vaccination side effects were reported.

All vaccinations were completed successfully, i.e., there were no cases in which the discomfort was so high that the vaccination had to be aborted.

### 3.2. VACS Calculation, Reliability and Validity

The weights presented in Section 2.3 were used to calculate VACS for each of the 40 recorded vaccinations. A first finding is that vaccination discomfort is not a problem for all children, with 16 (40%) of the vaccinations having a VACS score of exactly zero, i.e., the children did not display any indication of discomfort. The remaining children had VACS values ranging from 1 to 21 (median 6, mean 7.92, 95% CI:5.04–10.8). For the remainder of our study we have focused on the children that have non-zero VACS values, i.e., the children that have shown some discomfort during the vaccination procedure.

We quickly observe that the doctors considered ALL vaccinations with a VACS number below 19 as smooth and ALL vaccinations with a VACS number above 19 as merely acceptable. Vaccination procedures that are deemed smooth by the doctors produce lower VACS values and less smooth procedures produce larger VACS values.

The fact that VACS can perfectly differentiate between acceptable and not acceptable discomfort, from the point of view of the doctor, is a testament to the validity of the scale. Content, face and construct validity are given by the construction, due to the way VACS was designed, as it quantifies all the parameters and doctors empirically make observations in order to assess how children are coping with vaccinations.

The fact that the differentiation between acceptable and not acceptable discomfort holds true for all the doctors that participated in the clinical study is a testament to the reliability of the scale.

A by-product of this observation is that a threshold can be defined at a VACS value of 19, as the point above which vaccinations can no longer be deemed smooth.

### 3.3. Interesting Correlations

Unlike doctors, children start stating that they are unwilling to return for a future vaccination when their experience has produced a VACS value as low as 11. It is, we believe, an important finding as it highlights the disconnect between how children experience vaccination and how doctors consider children’s discomfort. Considering only the doctors’ point of view when specifying new vaccination procedures would set an insufficient target, as our finding shows that there are cases (VACS values between 11 and 19) in which the doctors estimate that the procedures are smooth, yet the children continue to suffer. Consequently, in order to effectively mitigate vaccination discomfort for children, we need a tool that assesses discomfort from the point of view of the children and a target threshold that is acceptable not only to doctors but to children as well.

VACS can be such a tool and, based on our findings, an even lower VACS threshold needs to be set as a target: whilst a threshold of 19 is sufficient for doctors to consider vaccinations below this threshold as acceptable, the threshold would have to be lowered to 10 to make sure that children are also content with their experiences.

One important question, of course, is that of predictability. If we can know beforehand which children are at a greater risk of experiencing discomfort, then perhaps we can focus more on how to best deal with them. Unfortunately, our data indicate that the parameters we have considered are poor predictors of VACS values.

**Figure 6:**
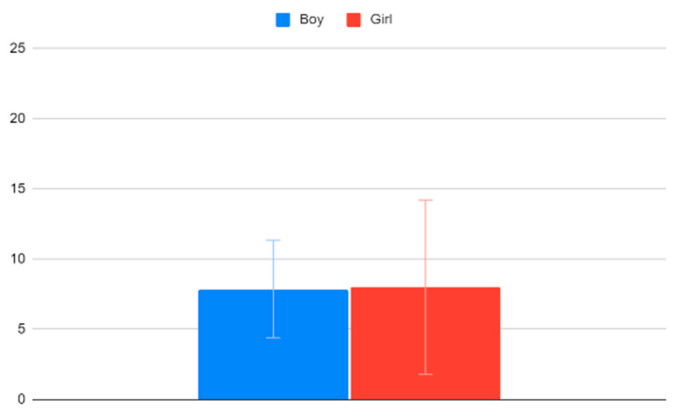
VACS values for boys and girls.

Boys have an average VACS of 7.87 (95% CI:4.4–11.33) and girls an average VACS of 8 (95% CI: 1.79–14.21). As also seen in Figure 6, there is no significant difference between the genders.

**Figure 7:**
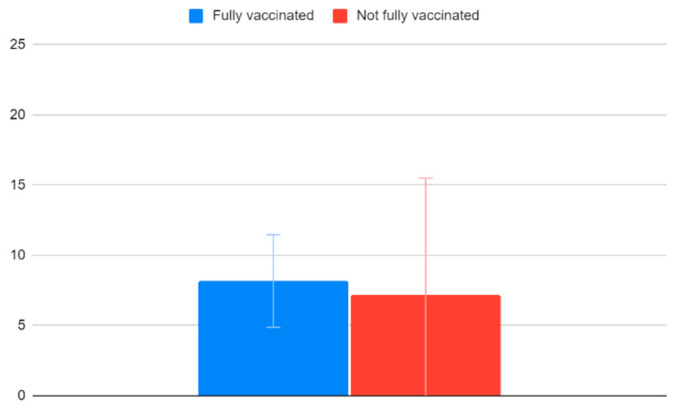
VACS values and vaccination history.

Children that have completed their recommended vaccination programs have an average VACS of 8.17 (95% CI: 4.86–11.47) and children who have not completed their recommended vaccination programs have an average VACS of 7.17 (95% CI: −1.16–15.49). As also seen in Figure 7, there is no significant difference based on whether the children are fully vaccinated.

**Figure 8:**
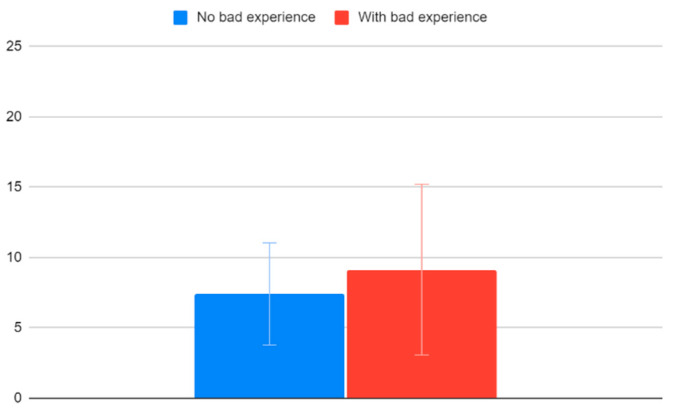
VACS values and bad vaccination experience history.

Children that have not had a previous negative vaccination experience have an average VACS of 7.41 (95% CI: 3.78–11.04) and children who have had a bad experience before have an average VACS of 9.14 (95% CI: 3.07–15.22). As also seen in Figure 8, although there is a small difference in the mean values, the confidence intervals are much wider, making this difference of no significance.

**Figure 9:**
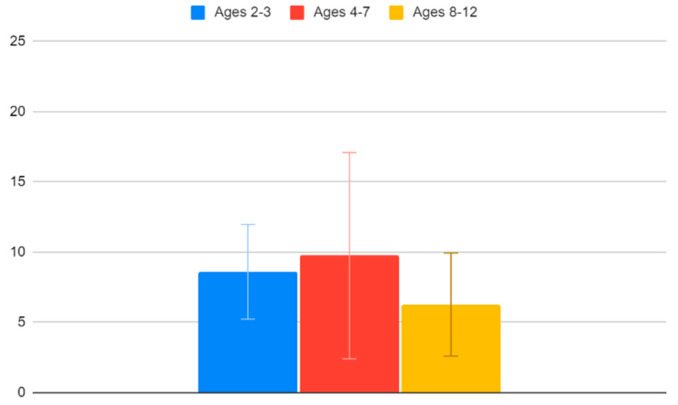
VACS values and age.

Children between the ages of 2 and 3 have an average VACS of 8.6 (95% CI: 5.23–11.97), children between the ages of 4 and 7 have an average VACS of 9.75 (95% CI: 2.41–17.09) and children between the ages of 8 and 12 have an average VACS of 6.27 (95% CI: 2.6–9.94). Unsurprisingly, we find that older children tend to cope better with the vaccination procedure. Again, though, as also seen in Figure 9, the confidence interval ranges are too broad to draw this conclusion safely.

## 4. Discussion

There are many cases in which simple numerical scales are used in medicine to acquire quick and rough assessments of situations. These scales are not necessarily fully descriptive of all aspects of the observed phenomenon, but they are sufficient to provide a first indication of how close the situation is to the “normal” or “safe” range and whether some intervention is required or not. Examples include APGAR for newborns [17], Childhood Autism Rating Scale (CARS) for autism [18], Abbreviated Mental Test score (AMTS) for dementia [19], Montgomery–Åsberg Depression Rating Scale (MADRS) [20] for depressive episodes and so on. The scale that we present in this work, VACS, aims to provide a similar tool for vaccination; it does not aim to measure discomfort in great detail, but rather to provide a quick and rough estimation of how smoothly a vaccination has been for a child.

The medical scales that are most similar and relevant to VACS are those that aim to measure pain, in the case of young children [16] and in the case of newborns [21]. However, pain is not the only source of discomfort for children during vaccination. It is probably not even the main one. This is evident by the fact that their discomfort often starts well before the actual vaccination shot, so it is clearly not induced only by pain. A metric viewing discomfort from a broader perspective and from the point of view of the children was needed; this is the gap that VACS aims to fill.

The fact that the majority of children display little or no discomfort is perhaps the reason that the alleviation of this discomfort has not been prioritized. We argue that this is not the medically prudent approach. As an exaggerated example, let us consider the case of poliomyelitis. Polio paralysis is much less frequent than severe vaccination discomfort. Less than 1% of poliovirus infections result in paralysis [22] compared to 15% of the vaccinations resulting in so much discomfort that the children are unwilling to be vaccinated again. Still, we consider polio as serious risk due to the severity of its complications on the rare occasions that they appear. In a somewhat similar line of thinking, even if only a minority of children feel discomfort during vaccination, this discomfort is important to these children and it is our duty to seek ways to minimize it. Moreover, even if only a small percentage of children experience intense vaccination discomfort, the fact that vaccination-age children constitute a quarter of the whole human population means that the number of suffering individuals is at least in the hundreds of millions. Acknowledging and measuring this discomfort—instead of simply ignoring it and focusing only on completing the vaccination—is a first step to reducing it.

### 4.1. Implications and Future Directions

The definition of VACS that is provided in this work can form the basis for further research and progress in the direction of comfortable vaccination. Having a tool with which to measure the discomfort experienced by children during vaccination, we can now use it in order to develop and then assess the effectiveness of alternative vaccination procedures. For example, a few years ago a VR system was presented for discomfort-free vaccinations of children [23], but to this day no evidence has been provided regarding its effectiveness. With VACS, the efficacy of such solutions can now be quantified. A clinical trial can be set up in which a control population is vaccinated conventionally and a similar test population is vaccinated using the VR system. The existence or absence of statistically important differences between the VACS measurements in the two populations can be used to objectively assess the effectiveness of the use of a VR system in the reduction in the vaccination discomfort.

Our team has already started to develop a proprietary VR system, similar to the one presented in [23], which we hope to assess using VACS and then release within the next couple of years.

Of course, high-tech VR solutions are not necessarily the only way to improve childrens’ vaccination experiences. Other interventions in the vaccination procedure, ranging from the way the vaccination room is organized to the background music and from the way the doctor talks to the child to the way the doctor is dressed, can now be examined and assessed. The efficacy of all and any of these in reducing children’s discomfort needs to be objectively assessed, via a clinical trial, such as the one described above for VR systems. Again, a discomfort measurement tool such as VACS is an essential component.

The fact that the parameters examined in our study (gender, previous negative vaccination experiences and whether a recommended vaccination program had been completed) were found to be poor predictors of vaccination discomfort does not mean that vaccination discomfort is impossible to predict. Instead, it highlights the need for further research, with the examination of more parameters. This is an important research direction, as identifying the characteristics of children who experience intense vaccination discomfort may be key to understanding the core routes of this discomfort and designing mitigating measures.

Finally, individual pediatricians can use VACS to assess their own vaccination procedure and performance. If they find that their average VACS value to be high, or that they have a high percentage of vaccinations that register VACS values above 10, perhaps there is a reason to re-assess how they approach the vaccination procedure.

### 4.2. Weaknesses

Whilst the new scale is clearly validated (low values coincide with children’s, parents’ and doctors’ views that the vaccination was smooth and extreme discomfort produces the highest values), the small size of the observed sample does not allow for a very detailed estimation of the thresholds with high confidence. When a much larger number of vaccinations has been carried out and recorded, it will be possible to estimate more reliably the thresholds of VACS values for truly smooth, acceptable and unbearable vaccinations.

The information regarding previous negative experiences and previous vaccination side effects does not come from medical records but rather from the parents’ subjective reporting. Whereas one parent might consider an experience to be okay and some side effects too minor to report, another parent might consider the same experience as negative and the same side effects as important. Therefore, a child’s historical data regarding previous experiences and side effects is not as reliable as the information observed by the doctors themselves.

Regarding the children’s opinion about their vaccination experience, it is more reliable for the older children. For children closer to the age of two, whose communication skills are still limited, their opinion is extracted and recorded with a much lesser certainty.

Finally, doctors’ assessments are also subjective and different doctors may assess the same observations differently. Whereas one doctor might record light moaning, another might note loud crying. As a result, VACS measurements are more reliably comparable only when they are performed by the same clinician; when different clinicians are involved, comparisons are safe only for larger differences between VACS measurements.

## 5. Conclusions

In this work we have presented VACS, a simple and easily applicable scale that measures the discomfort experienced by children aged 2–12 during the vaccination process. VACS measures discomfort as a number in the 0–25 range, with 0 corresponding to no discomfort whatsoever and 25 to maximal and totally intolerable discomfort. The scale’s validity has been established via a clinical study.

The clinical study has also allowed for the estimation of two critical thresholds. A VACS value of 11 is the threshold below which we can assume the child is having minimal or negligible discomfort whilst a VACS value of 19 is the threshold above which we can assume that the child is having an absolutely intolerable experience.

Our findings indicate that vaccination discomfort is an issue of varying gravity for approximately 60% of children. The demographics and parameters considered in our research, such gender, whether the state’s recommended vaccination program has been implemented in full and even prior negative vaccination experiences are not correlated with the VACS measurements of subsequent publications; age on the other hand may be correlated to VACS measurements, but further research is required to establish this with reasonable confidence.

The establishment of VACS opens the way for further progress in a range of directions. On one hand, pediatric clinics and individual clinicians can measure their performance against the identified thresholds in order to assess the quality of experiences they offer to their patients. On the other hand, those designing and developing methods and systems for the vaccination of minors can use VACS in order to assess the efficiency of their approaches. In both cases the main contribution of VACS is that these assessments, that until today could only be performed in a qualitative and subjective manner, can now be performed in a quantitative and objective way.

## Figures and Tables

**Table 1:** Distribution of VACS parameter weights in the four stages.

Stage	Maximum Points	Percentage
Stage I—Entry	5	20%
Stage II—Examination	8	32%
Stage III—Procedure	8	32%
Stage IV—Completion	4	16%

**Figure A1:**
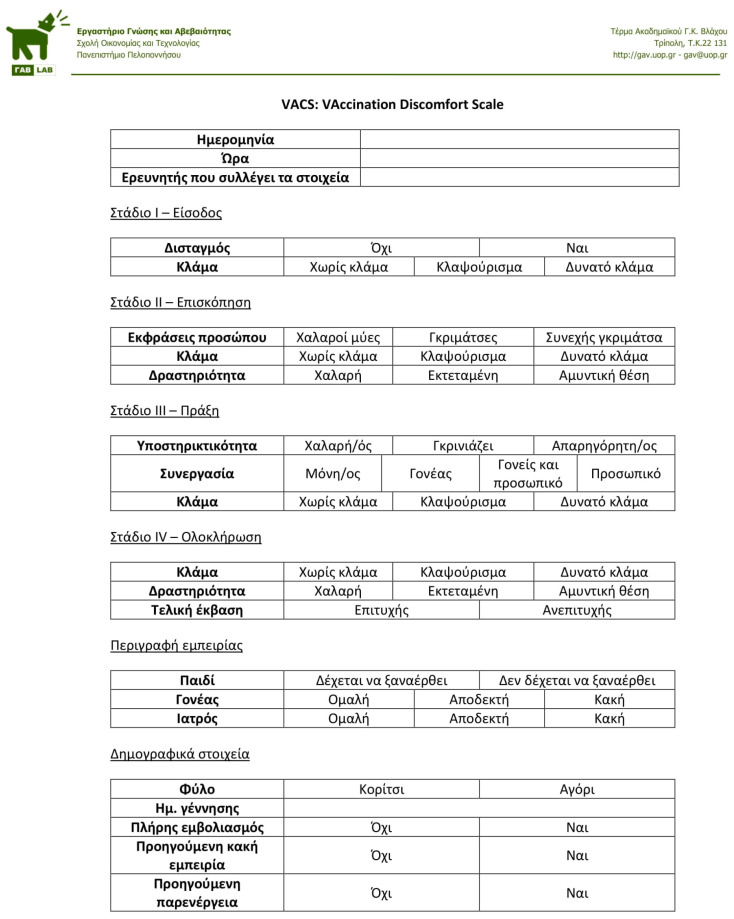
The form used to collect the data.

**Figure A2:**
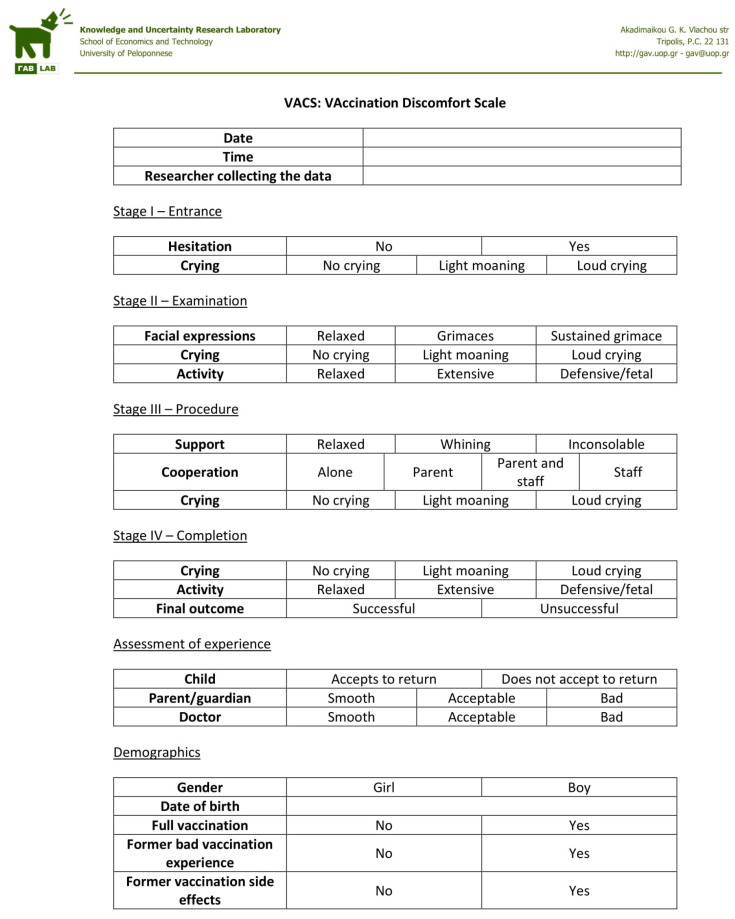
The form used to collect the data, translated into English.

## Data Availability

The data presented in this study are available upon reasonable request from the corresponding author. The data are not publicly available due to their nature as they include personal records of individual minors.

## Appendix A

In the following images we present the form used to collect the data upon which the VACS calculation is based. We provide an English translation of the form, for international pediatricians who wish to incorporate VACS into their own clinical practice.
